# Patients with clinically diagnosed hand OA not fulfilling the ACR classification criteria are in an earlier disease phase and more often have thumb base OA

**DOI:** 10.1016/j.ocarto.2023.100347

**Published:** 2023-02-18

**Authors:** Sjoerd van Beest, Lotte A. van de Stadt, Frits R. Rosendaal, Margreet Kloppenburg

**Affiliations:** a Department of Rheumatology, Leiden University Medical Center, Leiden, the Netherlands; b Department of Clinical Epidemiology, Leiden University Medical Center, Leiden, the Netherlands

**Keywords:** Hand osteoarthritis, Classification criteria, Short Form-36, Australian/Canadian hand OA index, Radiography

## Abstract

**Objective:**

To investigate the performance of the American College of Rheumatology (ACR) classification criteria for hand osteoarthritis.

**Design:**

Longitudinal data up to four years from a cohort of consecutive patients with primary hand osteoarthritis diagnosed by their rheumatologist (Hostas study) were used to classify presence or absence of hand osteoarthritis according to the 1990 ACR criteria (traditional format: one major and 4 minor ACR criteria) (ACR^+^/ACR^−^). Demographics, Australian/Canadian osteoarthritis hand index (AUSCAN) pain and function were obtained. Hand radiographs were scored according to Kellgren-Lawrence; radiographic osteoarthritis was defined as Kellgren-Lawrence ≥2 in ≥1 CMC1 joint or ≥2 DIP/PIP/MCP joints.

**Results:**

Of 538 patients (mean age 61 years, 86.1% women) 485 (90.1%) fulfilled ACR criteria at baseline. Except for the minor criterion swelling of <3 MCP joints, all criteria differed between the groups. ACR^−^ patients were younger, with higher BMI, a shorter time since diagnosis, and less bony enlargements, joint deformities and radiographic osteoarthritis, except for radiographic CMC1 osteoarthritis which was seen more often in ACR^−^ patients. No difference in AUSCAN pain or function was seen between ACR^−^ versus ACR^+^ patients. After follow-up 37/53 (69.8%) converted to ACR^+^, 2/53 (3.8%) did not, and 14/53 (26.4%) were lost to follow-up.

**Conclusions:**

In clinical practice the majority of patients fulfill the ACR classification criteria, but those in an earlier disease phase, with less signs of hand osteoarthritis or with primarily thumb base osteoarthritis are less likely to fulfill them. New classification criteria also including earlier disease stages and with attention for hand osteoarthritis subtypes are required.

## 1 Introduction

Hand osteoarthritis (OA) is a painful, disabling group of disorders, characterized by hard tissue enlargements and deformities of the hand joints, resulting in a decreased quality of life [1]. It is highly prevalent, especially in women, with an estimated overall lifetime risk of symptomatic hand OA of 39.8% in a North-American population [2]. Currently, knowledge of pathogenesis and development of hand OA is limited, and also treatment options are not sufficient in many patients. Given the disease impact and the limited research data obtained until now, further research to improve insight in hand OA is required.

Hand OA especially affects the finger joints, including the distal interphalangeal (DIP), proximal interphalangeal (PIP) and 1st interphalangeal (IP1) joints, next to the thumb base joints, including the 1st carpometacarpal (CMC1) and scaphoid-trapezium-trapezoid joints, which can occur together or apart [3,4]. Hand OA involves different joints over time, with intercurrent symptoms and signs, as pain, redness, and soft tissue swelling, and with gradually development of bony enlargements and deformities of affected joints. Consequently, an early stage in one joint can occur at the same time as a late stage in another joint. Moreover, thumb base OA causes relatively more symptoms although it affects less joints than finger OA [5], and seems to have another underlying pathogenesis than finger OA [[6], [7], [8]]. The different possible combinations of involved hand joints make hand OA a heterogeneous disorder that is complex to study.

The present classification criteria that were developed to aid in studies concerning hand OA were developed in 1990 by the American College of Rheumatology (ACR) to classify hand OA [9]. These criteria, based on symptoms and clinical signs, don't differentiate between finger and thumb base OA. Moreover, they were developed by comparing patients with hand OA to patients with inflammatory arthritides, such as rheumatoid arthritis, spondylarthritis and gout. The sensitivity and specificity of these criteria were reported to be good (sensitivity 94% and specificity 87%) [9]. But how these criteria perform in other populations is not known.

In the present study we investigated whether patients from a rheumatology outpatient clinic diagnosed by their treating rheumatologist with hand OA fulfill the ACR classification criteria and what the characteristics are of those patients that have the clinical diagnosis of hand OA, but do not classify as such.

## 2 Methods

### 2.1 Study population

We used longitudinal data from the ongoing Hand OSTeoArthritis in Secondary care (HOSTAS) observational cohort study, in which consecutive patients diagnosed with primary hand OA by their treating rheumatologist were included between 2009 and 2015 [10]. Upon entering a patient in the study, the rheumatologists were asked to grade the level of certainty they had in the given diagnosis on a numerical rating scale 0–10 (higher is more certain). Exclusion criteria were: any other pathological condition explaining the hand symptoms, i.e. inflammatory arthropathies or fibromyalgia, and secondary OA, e.g. due to hemochromatosis. Written informed consent was obtained from all participants. The study was approved by the Leiden University Medical Center medical ethics committee.

### 2.2 Clinical examination

Demographics and clinical characteristics were collected by standardized questionnaires. Education level was divided into 3 groups: low (no education, primary school only, lower vocational education), middle (lower general secondary, secondary vocational education), and high (all higher education). Presence of hand pain, aching or stiffness, osteoporosis and comorbidities from the modified Charlson index [11,12] were assessed by questionnaire and verified by research nurses. In addition, the severity of self-reported hand symptoms was assessed by the Australian/Canadian hand OA index (AUSCAN) subscales pain, stiffness and function. Height and weight were measured, to calculate body mass index (BMI). Quality of life was assessed by the Dutch Research and Development (RAND) translation [13] of the Short Form Health Survey (SF-36) [14]. Physical and mental component summary scales (PCS and MCS, respectively) were calculated using norm based population data resulting in standardized scores: range 0–100, mean 50, standard deviation 10, and lower score is worse health [15]. Trained research nurses performed the physical examination, assessing the DIP, PIP, IP1, metacarpophalangeal (MCP) and CMC1 joints of both hands for the presence of soft tissue swelling, hard tissue enlargement and deformity. Also patient's crepitus, bony tenderness, bony enlargement, and palpable warmth of the knee and internal rotation of the hip were examined. Questionnaires were repeated annually and follow-up visits with physical examination were repeated two and four years after baseline.

### 2.3 Radiography

Baseline dorsopalmar radiographs of both hands were scored 0–4 following Kellgren-Lawrence (KL) scoring, assessing the DIP, PIP, IP, MCP and CMC1 joints (30 joints per patient) [16]. A single reader (WD) scored all radiographs, blinded for demographic and clinical data, with high intraobserver reliability [17]. Patients with at least one CMC1 joint or at least two other hand joints with a KL-score ≥2 were considered to have radiographic HOA.

### 2.4 Statistical analysis

Fulfilment of the ACR classification criteria for clinical hand OA [9], hip OA [18] and knee OA [19], was determined for all patients at baseline. Presence of hand OA was defined following the “traditional format” as the presence of pain, aching or stiffness (major criterion), and the presence of ≥3 of 4 minor criteria, being hard tissue enlargement of ≥2 of 10 selected joints, hard tissue enlargements of ≥2 DIP joints, <3 swollen MCP joints, and deformity of ≥1 of 10 selected joints. The selected joints are the 2nd and 3rd DIP and PIP joints, and the CMC1 joints. Presence of hip or knee OA was defined as either fulfilling the clinical classification criteria or having a prosthesis. Based on the ACR criteria for clinical hand OA, we split the cohort in two groups, comparing patient characteristics, imaging scores and clinical outcome measures at baseline between patients fulfilling the criteria (ACR^+^) and patients not fulfilling the criteria (ACR^−^) using Student T-tests, Pearson Chi-Square tests, or Mann-Whitney U tests when appropriate. Additionally, we generated cumulative probability plots for the number of joints with hard tissue enlargement, AUSCAN pain and AUSCAN function scores (higher scores indicate worse health), and heatmaps showing the relative distribution of joints with radiographic OA (KL-score ≥2) to enable a visual comparison between ACR^+^ and ACR^−^ patients.

Next, we plotted an area-proportional Euler diagram with ellipses [20] showing which of the following ACR criteria were not met: hand pain, aching or stiffness (major criterium [M]), hard tissue enlargement of at least two out of ten selected joints (minor criterium 1 [m^1^]), hard tissue enlargement of at least two DIP joints (m^2^), fewer than three swollen MCP joints (m^3^), and deformity of at least one out of ten selected joints (m^4^). The forementioned ten selected joints are the DIP and PIP joints of left and right index and middle finger and both CMC1 joints. In the ‘traditional format’ application of these criteria, a patient is classified positive when the major criterium and at least three minor criteria are present [9]. Therefore, in this diagram the absent major criterium or the intersection of two or more absent minor criteria depict the proportion of ACR^−^ patients. The second method of applying these criteria, based on a classification tree as described in the original article [9], was not used in this study. For patients not fulfilling the major criterium, we checked the reason for consultation in the referral letter from the general practitioner.

Follow-up data from ACR^−^ patients was used to re-evaluate fulfillment of the criteria that were not met at baseline. Positive major and minor criteria at baseline were not re-evaluated at follow-up and were considered to remain positive. Likewise, ACR^+^ patients – also those who only fulfilled three minor criteria – were not re-evaluated. The percentage of ACR^+^ patients in Hostas over time was calculated as cumulative survival function without censoring ACR^−^ patients who left the study before fulfilling the criteria.

A second area-proportional Euler diagram was drawn based on the cumulative data after four years of follow-up, according to the same rules as at baseline. From the ACR^−^ patients at baseline, we compared patient characteristics, imaging scores and clinical outcome measures between those who became ACR^+^ and those who were still ACR^−^ or had stopped participating.

Data were analysed using SPSS for Windows, version 26.0 (IBM, New York, USA). Area Proportional Euler diagrams were drawn using eulerAPE, version 3.0.0 (freely available at http://www.eulerdiagrams.org/eulerAPE/) [20].

We followed the ‘Strengthening the reporting of observational studies in epidemiology’ (STROBE) guidelines for transparent reporting of observational studies [21].

## 3 Results

### 3.1 Study population

During the study recruitment period, 629 patients were consecutively diagnosed with hand OA, of whom 80 did not consent to participate and 11 fulfilled exclusion criteria after medical chart verification. The majority of the included 538 patients were middle-aged and female. Typically, patients suffered from hand OA symptoms for several years before visiting the rheumatology outpatient clinic and many also had OA of the lower extremities (Table 1). Data were complete for age, sex, and physical examination. Less than 3% of data was missing for other variables, except SF-36 (5%), symptom duration (7%), rheumatologist's certainty in diagnosis rating (10%) and time since hand OA diagnosis (12%). Patients with complete data for all variables in Table 1 did not differ from the total population (not shown).

**Table 1:** Baseline characteristics of 538 hand OA patients in the HOSTAS cohort.

Age (years)	61.0 (8.6)
Women, n (%)	463 (86.1)
BMI (kg/m^2^)	26.2 (23.7–29.6)
Employment status, n (%):	
- Working	215 (41.2)
- Pension	161 (30.8)
- Sickness leave/work disabled	67 (12.8)
- Unemployed/other	79 (15.2)
Education level, n (%):	
- Low	141 (26.9)
- Middle	204 (38.9)
- High	179 (34.2)
Any comorbidity present, n (%)	205 (38.6)
Number of comorbidities	0 (0–1)
Generalized OA, n (%)	
Knee and/or hip OA	227 (43.2)
Knee OA	197 (37.3)
Hip OA	58 (11.0)
**General and disease-specific burden**	
Self-reported health-related quality of life	
PCS	45.2 (39.1–50.8)
MCS	53.9 (48.5–57.5)
AUSCAN pain (0–20)	9.5 (6–12)
AUSCAN stiffness (0–4)	2 (1–2)
AUSCAN function (0–36)	16 (9–22)
AUSCAN total score (0–60)	27 (17–36)
**Hand-specific disease characteristics**	
Symptom duration (years)	5.3 (1.9–12.3)
Time since diagnosis (months)	2.3 (0.9–22.8)
Radiography	
Summated KL-scores (0–120)	17 (8–29)
KL-score ≥2 joint count (0–30)	4 (1–9)
HOA phenotype, n (%)∗:	
- No radiographic HOA	148 (27.7)
- Radiographic IPJOA, without TB involvement	159 (29.8)
- Radiographic TBOA, without IPJ involvement	38 (7.1)
- Radiographic HOA, affecting both TB and IPJ	189 (35.4)

Mean (SD) or median (IQR) unless stated otherwise.

∗ Radiographic HOA was defined as a KL-score ≥2 of at least one CMC1 joint or at least two other hand joints. AUSCAN, Australian/Canadian osteoarthritis hand index; BMI, body mass index; HOA, hand osteoarthritis; HOSTAS, Hand OSTeoArthritis in Secondary care; IPJ, interphalangeal joint; KL-score, Kellgren-Lawrence osteoarthritis grading scale; MCS, mental component summary scale; PCS, physical component summary scale; TB, thumb base.

### 3.2 Fulfilment of the ACR classification criteria for clinical hand OA

**Fig. 1:**
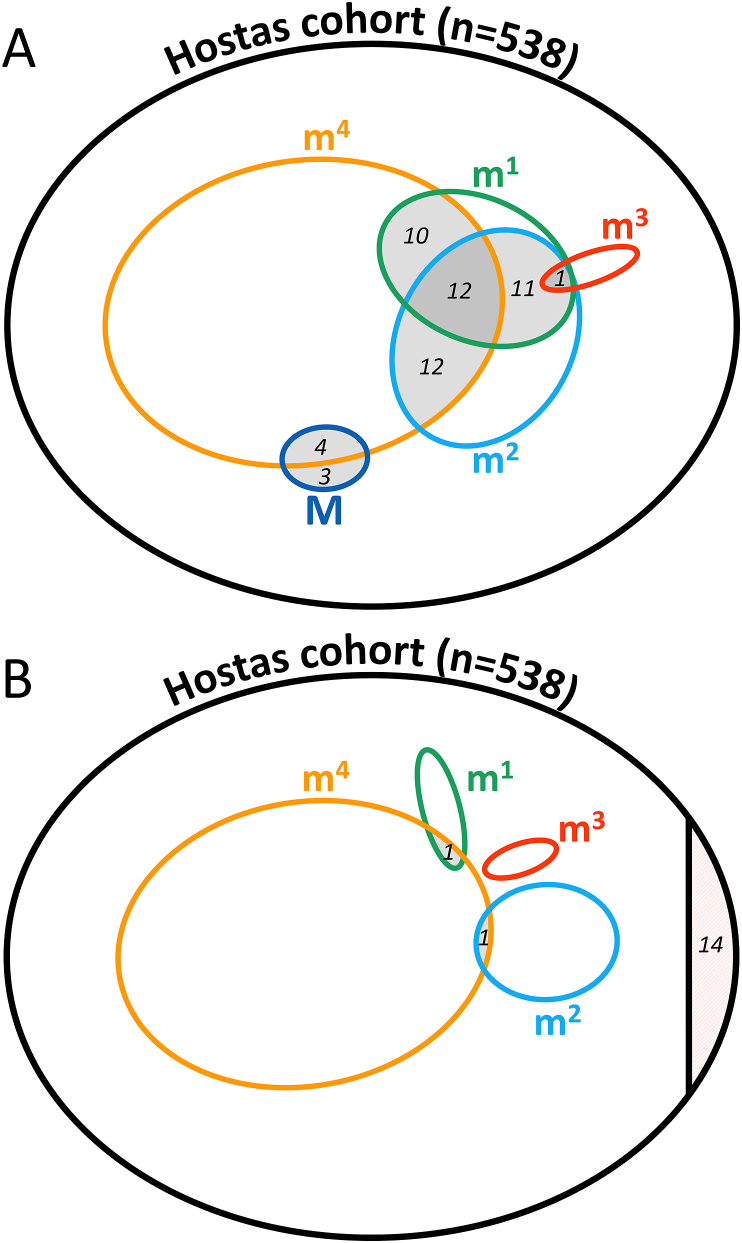
Area proportional Euler diagrams depicting the number of patients not fulfilling each of the criteria: hand pain, aching or stiffness (major criterion [M], *dark blue*), hard tissue enlargement of at least two out of ten selected joints (minor criterion 1 [m^1^], *green*), hard tissue enlargement of at least two DIP joints (m^2^, *light blue*), fewer than three swollen MCP joints (m^3^, *red*), and deformity of at least one out of ten selected joints (m^4^, *yellow*) from the total of 538 patients (*black*) at baseline (A) and four-year follow-up (B). An absent major criterion or the intersection of two or more absent minor criteria depicts the proportion of ACR^−^ patients (*shaded grey*). ACR^−^ patients who left the study before fulfilling the criteria are *shaded red* (n ​= ​14) (B). (For interpretation of the references to color in this figure legend, the reader is referred to the Web version of this article.)

Out of 538 hand OA patients 485 (90.1%) fulfilled the ACR criteria for hand OA at baseline (ACR^+^). The majority of patients not fulfilling the criteria (ACR^−^) did fulfill the major criterium (Table 2), but had two or more absent minor criteria (Fig. 1a). For the patients not fulfilling the major criterium (n ​= ​7), the reason for consulting a rheumatologist was obviously not the presence of hand pain or stiffness. However, six patients had had hand pain or stiffness in the past, but developed polyarticular OA and now consulted the rheumatologist for OA symptoms at other locations. Only one patient did not report any pain or stiffness, but worried over disfigurement of her fingers due to Heberden's noduli and flexion-deformities of multiple DIP joints.

**Table 2:** Fulfilment of each criterion from the ACR classification criteria for clinical hand OA at baseline compared between patients fulfilling the criteria (ACR^+^) and patients not fulfilling the criteria (ACR^−^) in the HOSTAS cohort (n ​= ​538).

	ACR^+^ (n ​= ​485)	ACR^−^ (n=53)	*p* ​=
Major criterium (hand pain, aching or stiffness), n (%)	485 (100)	46 (86.8)	<0.001
Minor criteria, n (%)			
Hard tissue enlargement ≥2 of 10 selected joints^a^	481 (99.2)	19 (35.8)	<0.001
Hard tissue enlargement ≥2 DIPJs	469 (96.7)	17 (32.1)	<0.001
Swelling <3 MCPJs	482 (99.4)	52 (98.1)	0.340
Deformity ≥1 of 10 selected joints^a^	364 (75.1)	15 (28.3)	<0.001

a Ten selected joints are left and right DIP2, PIP2, DIP3, PIP3 and CMC1 joints. ACR, American College of Rheumatology; CMC1, first carpal-metacarpal; DIP(J), distal interphalangeal (joint); HOSTAS, Hand OSTeoArthritis in Secondary care; MCPJ, metacarpal-phalangeal joint; PIP, proximal interphalangeal.

Joint deformity was the least present minor criterium in both ACR^+^ and ACR^−^ patients, whereas almost all patients fulfilled the third – less than three soft swollen MCPs – minor criterium (99.3%). Leaving out the latter would not have made any difference in the final fulfilment of the ACR criteria (Fig. 1a).

### 3.3 Baseline differences between ACR^+^ and ACR^−^ patients

The rheumatologists’ certainty in the hand OA diagnosis was high in both groups, although the certainty in the ACR^+^ patients was even higher (median 9 (interquartile range 8–10), n ​= ​435) than in the ACR^−^ patients (8 (7–9), n ​= ​50) (*p* ​= ​0.011).

Baseline characteristics were compared between ACR^+^ and ACR^−^ patients (Table 3).

**Table 3:** Baseline characteristics compared between patients fulfilling the criteria (ACR^+^) and patients not fulfilling the criteria (ACR^−^) in the HOSTAS cohort (n ​= ​538).

	ACR^+^ (n ​= ​485)	ACR^−^ (n=53)	*p* ​=
Age (years)	61.4 (8.6)	57.4 (8.2)	0.001
Women, n (%)	420 (86.6)	43 (81.1)	0.295
BMI (kg/m^2^)	26.1 (23.6–29.4)	28.0 (25.1–32.3)	0.015
Employment status, n (%):			0.104
- Working	191 (40.5)	24 (48.0)	–
- Pension	153 (32.4)	8 (16.0)	–
- Sickness leave/work disabled	57 (12.1)	10 (20.0)	–
- Unemployed/other	71 (15.0)	8 (16.0)	–
Any comorbidity present, n (%)	185 (38.5)	20 (39.2)	0.925
Number of comorbidities	0 (0–1)	0 (0–1)	0.889
Generalized OA, n (%)			
Knee and/or hip OA	200 (42.3)	27 (51.9)	0.188
Knee OA	171 (35.9)	26 (50.0)	0.051
Hip OA	55 (11.6)	3 (5.7)	0.249
**General and disease-specific burden**			
Self-reported health-related quality of life			
PCS	45.4 (39.2–50.9)	42.0 (36.6–48.6)	0.068
MCS	53.8 (48.2–57.4)	54.7 (49.2–59.0)	0.234
AUSCAN pain (0–20)	9 (6–12)	10 (6–13)	0.736
AUSCAN stiffness (0–4)	2 (1–2)	2 (1–2.25)	0.872
AUSCAN function (0–36)	16 (9–22)	16 (9–22)	0.974
AUSCAN total score (0–60)	27 (17–35)	26.5 (18–37)	0.953
**Hand-specific disease characteristics**			
Symptom duration (years)	5.4 (2.0–12.3)	4.0 (0.9–11.7)	0.123
Time since diagnosis (months)	2.5 (0.9–24.5)	1.6 (0.7–3.5)	0.036
Radiography			
Summated KL-scores (0–120)	18 (10–31.5)	7 (3.5–14.5)	<0.001
KL-score ≥2 joint count (0–30)	5 (2–10)	1 (0–3)	<0.001
HOA phenotype, n (%)∗:			<0.001
- No radiographic HOA	122 (25.4)	26 (49.1)	–
- Radiographic IPJOA, without TB involvement	156 (32.4)	3 (5.7)	–
- Radiographic TBOA, without IPJ involvement	23 (4.8)	15 (28.3)	–
- Radiographic HOA, affecting both TB and IPJ	180 (37.4)	9 (17.0)	–

Mean (SD) or median (IQR) unless stated otherwise.

∗ Radiographic HOA was defined as a KL-score ≥2 of at least one CMC1 joint or at least two other hand joints. AUSCAN, Australian/Canadian osteoarthritis hand index; BMI, body mass index; HOA, hand osteoarthritis; HOSTAS, Hand OSTeoArthritis in Secondary care; IPJ, interphalangeal joint; KL-score, Kellgren-Lawrence osteoarthritis grading scale; MCS, mental component summary scale; PCS, physical component summary scale; TB, thumb base.

### 3.4 Patient characteristics

The ACR^−^ group was on average four years younger than ACR^+^ group and had a higher BMI. Furthermore a lower number of patients was retired, however the distribution over the employment status categories did not significantly differ between both groups. Knee OA appeared to be more prevalent, although not statistically significant (Table 3). Also after stratifying or adjusting for knee OA presence, BMIs remained significantly higher in the ACR^−^ group (data not shown). Education levels did not differ between the groups (data not shown).

### 3.5 Symptoms

**Fig. 2:**
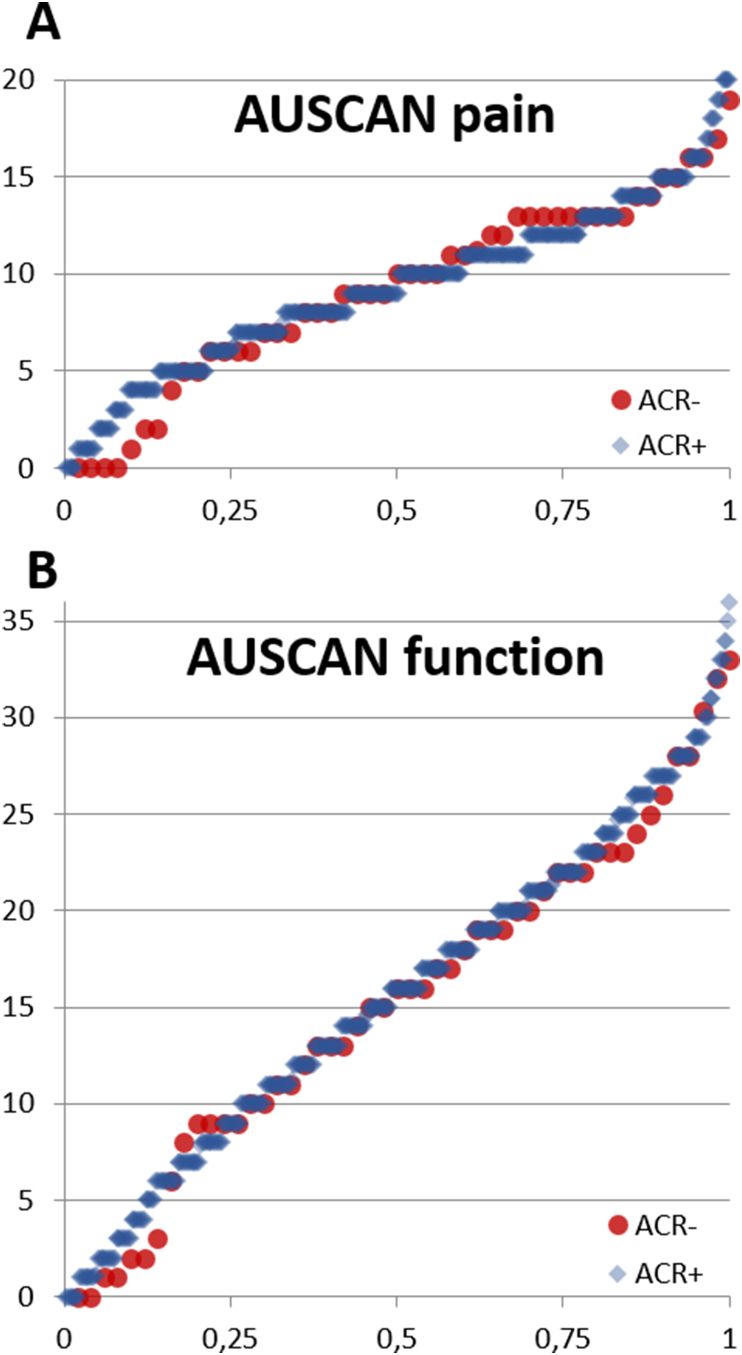
Cumulative probability plot showing the distribution of AUSCAN pain (A) and function scores (B) at baseline stratified for ACR^+^ (*blue diamond*) and ACR^−^ (*red dot*) patients. (For interpretation of the references to color in this figure legend, the reader is referred to the Web version of this article.)

Although not reaching statistical significance, ACR^−^ patients scored worse on the physical component summary scale for norm-based self-reported health-related quality of life. The presence and severity of hand pain, stiffness and functional limitations varies greatly between patients, however AUSCAN scores were similarly distributed for ACR^+^ and ACR^−^ patients (Table 3, Fig. 2). Also symptom duration varied greatly between patients and ACR^−^ patients usually had experienced symptoms for many years, which was not different from ACR^+^ patients. Time since diagnosis, which could be made by a general practitioner, treating rheumatologist, or other physician earlier consulted, was shorter in ACR^−^ patients.

### 3.6 Signs

**Fig. 3:**
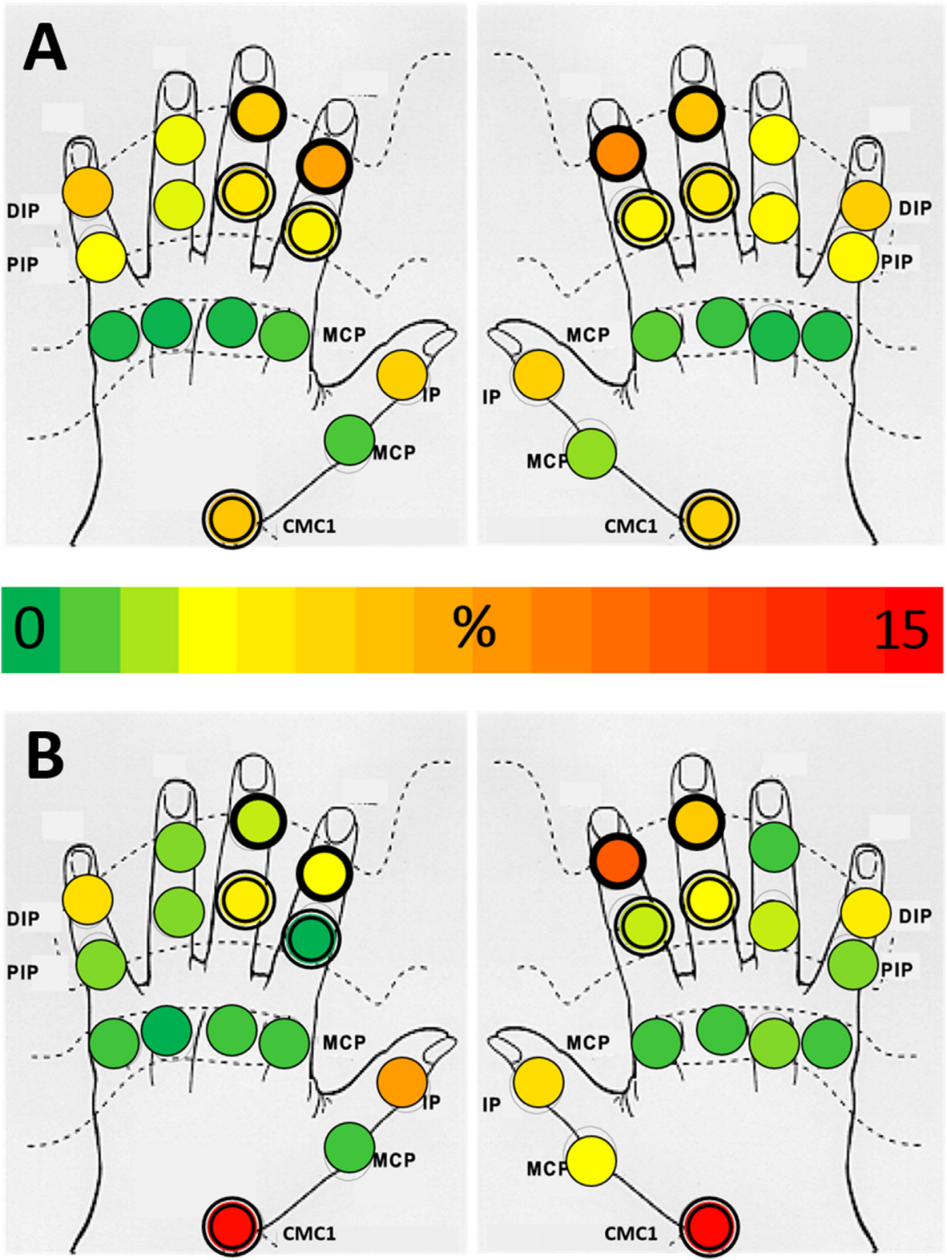
Heat map of relative distribution of joints with radiographic OA (KL-score ≥2) at baseline for ACR^+^ (A) and ACR^−^ (B) patients. Joints encircled by double line (PIP2, PIP3, CMC1) are the subject of two minor criteria; joints encircled by thick solid line (DIP2, DIP3) are the subject of three minor criteria. CMC1, first carpal-metacarpal (joint); DIP, distal interphalangeal (joint); IP, interphalangeal (joint); MCP, metacarpal-phalangeal (joint); PIP, proximal interphalangeal (joint).

Hard tissue enlargements of specific joints are the subject of the two criteria with the biggest difference between ACR^+^ and ACR^−^ patients (Table 2). Moreover, the total number of joints with hard tissue enlargement was much higher in the ACR^+^ group (85% of patients had 7 or more hard tissue enlarged joints) than in the ACR^−^ group (80% of patients had 6 or less hard tissue enlarged joints) (Supplementary Fig. 1). On hand radiographs of ACR^+^ patients the summated KL-score and the number of joints with KL-score ≥2 was significantly higher than on hand radiographs of ACR^−^ patients (Table 3). Furthermore, in the ACR^+^ group joints with radiographic OA (i.e. KL-score ≥2) were distributed symmetrically between the left and right hand, and all interphalangeal joints were commonly affected with a slight predisposition towards the second and third DIP joints; whereas in ACR^−^ patients the distribution was skewed towards the right hand, the fourth digit and PIP5 were rarely affected, and the thumb bases were more frequently involved (Fig. 3).

Although the difference in patients with radiographic thumb base OA between both groups is only three percentage point, it is less likely that a patient fulfills the ACR criteria when besides the thumb base no interphalangeal joints are involved (Table 3).

### 3.7 Change in ACR criteria status

We used follow-up data of the 53 patients who were ACR^−^ at baseline to re-evaluate their ACR criteria. After four years 37 patients had converted to the ACR^+^ group, of whom 27 already had converted after two years. Unfortunately 10 patients dropped out of the study before any re-evaluation, and an additional 4 patients that were still ACR^−^ at the two-year follow-up stopped before the four-year visit. Reasons for stopping before re-evaluation were: favorable symptom discourse (n ​= ​2), satisfactory treated with bilateral trapeziectomy (n ​= ​1), patient has deceased (n ​= ​1), because no intervention was offered (n ​= ​1), unknown (n ​= ​5). After four years only two patients actively participating in the HOSTAS study remained ACR^−^ (Fig. 1b): one patient is without any radiographic signs of OA, whereas the other patient's already has clear signs of (thumb base) OA on hand radiographs at the four-year visit.

The only difference at baseline between ACR^−^ patients that converted to ACR^+^ and those stopping or remaining ACR^−^ was a significantly higher body mass index for the latter (Supplementary Table 1).

## 4 Discussion

We applied the ACR classification criteria on a large cohort of patients diagnosed with primary hand OA by their treating rheumatologist from a secondary and tertiary clinic. Virtually all studies on clinical hand OA from the past two decades report the percentage of participants who meet the ACR classification criteria [[22], [23], [24]] or they might even use it as a study inclusion criterion [25,26], as is also recommended by the OARSI guideline for the conduct of clinical trials [27]. However, this study is the first to focus on patients who initially do not meet the ACR classification criteria, and assess how they differ from those that do meet the criteria at baseline and whether they develop classified hand OA over time.

Of all patients diagnosed by a rheumatologist around 10% did not meet the 1990 ACR classification criteria for hand OA. Among the ACR^−^ patients not all had pain, aching or stiffness, at time of assessment, which is required to be classified anyway. The question is whether this major criterion should always be required. For clinical trials investigating a symptom modifying drug the presence of pain or a pain phenotype is warranted. However, when extensive structural hallmarks of hand OA are present, but patients primary complaint is functional disability without pain, aching or stiffness, also a diagnosis of hand OA can be present, and such a patients could be included in a structure modifying trial. Remarkable, was that the criterion “fewer than 3 swollen MCP joints” was met by almost everybody in both groups. The question arises what the additive value of this criterion is? This criterion especially reflects the development process of the classification criteria where the control group consisted for the majority of patients with rheumatoid arthritis. All other minor criteria were less present in the ACR^−^ than in ACR^+^ groups, which indicates that the treating rheumatologist has a lower threshold to make a diagnosis of hand OA than based upon the classification criteria when less structural signs are present in the selected joints. Rheumatologists felt highly certain of the hand OA diagnosis in both groups.

We showed that at baseline ACR^−^ patients were on average four years younger than ACR^+^ patients, which suggests that these patients are in an early disease stage and might therefore not yet fulfill enough minor criteria. That ACR^−^ patients are in an earlier disease stage is further underscored by the difference in time since diagnosis, with ACR^+^ patients having received a prior hand OA diagnosis up to several years before entering our study. ACR^−^ patients also had significantly higher body mass index, which was independent from the higher knee OA prevalence. They had less frequent radiographic hand OA. But when ACR^−^ patients did have structural damage: the KL-scores were lower and fewer joints were involved. Moreover, relatively more often the thumb base joints were involved or joints that do not belong to the group of selected joints. Finally, the ACR^−^ patients had a higher BMI, than the ACR^+^ patients. One could wonder whether excess body mass might hinder the physical examination of hand joints for hard tissue enlargements. A study comparing physical examination and musculoskeletal ultrasound in rheumatoid arthritis patients showed that for higher BMI categories swollen joints were more often misclassified [28]. This was supported by the longitudinal data, since body mass index seemed to be the only discriminating factor at baseline between those who converted to ACR^+^ and those that remained ACR^−^ or discontinued the study.

Nevertheless, ACR^−^ patients experienced similar levels of hand pain, stiffness and function loss as ACR^+^ patients. The high disease burden of ACR^−^ patients was also supported by the low score on the physical component summary scale for norm-based self-reported health-related quality of life. This score tended to be even lower for ACR^−^ patients, and although the 3.4 median score gap with ACR^+^ patients did not constitute a statistical significant difference, it still might be a relevant result considering the reported minimal clinically important difference (MCID) of 2 [29]. Possible explanations might be the added suffering from the higher prevalence of knee OA or alternatively the younger age with a presumably more active lifestyle requiring a higher physical well-being and therefore a greater perceived loss of physical health. Appreciate that the norm-based score is weighted for age groups with ten year intervals [15], therefore not completely precluding the possible effect of an age difference.

With time going by the majority of ACR^−^ patients converted to ACR^+^ of whom many already during the first two years. This further supports the explanation that ACR^−^ patients were in an earlier disease stage at baseline than the ACR^+^ patients. However, one ACR^−^ patient discontinued the study after a satisfactorily treatment with bilateral trapeziectomy. Such a patient definitely has clinical hand OA, but does not meet the current classification criteria.

There are some limitations we need to address. First, there was a substantial drop out of ACR^−^ patients before they converted to ACR^+^ patients. Unfortunately, most patients who were lost to follow-up did not provide us with a reason for dropping out, however we are opined that by not censoring we have chosen a conservative approach. Second, as mentioned above, our findings suggest that ACR^−^ patients might be at an earlier stage of hand OA, however many patients with hand OA, and especially those in the earliest stages of the disease, will first seek medical attention in primary care, and are therefore not included in our cohort consisting of secondary and tertiary care patients. Third, patients had follow-up visits with physical examination only biennially. Therefore, conversion from ACR^−^ to ACR^+^ as a result of meeting more minor classification criteria could only be confirmed at the two and four year follow-up.

In conclusion, hand OA is a heterogeneous disease and the current ACR classification criteria are too restrictive, causing patients in an early stage of the disease or with a distribution of affected joints not pertaining to the selected joints in the criteria, to be left out when the criteria would be strictly applied. However, the hand OA research field would fare well with new, good classification criteria, which is why the EULAR taskforce for evidence-based recommendations identified the development of new classification criteria as a top research priority [30].

Because of the heterogeneous nature of hand OA, it might be difficult to create all-inclusive classification criteria. Instead the developers of new criteria should aim at creating separate subsets, for interphalangeal joint OA and for thumb base OA, for example. Likewise, as in the Spondylo Arthritis International Society (ASAS) classification criteria for axial spondyloarthritis, a clinical arm and an imaging arm could co-exist, as two separate ways to classify patients for the same group [31].

Currently, an international team of experts in the field of hand OA already is making good progress with the development of new hand OA classification criteria [32,33].

## Contributions

Conception and design of the study (SvB, MK), acquisition of data (SvB, MK), analysis (SvB, LAvdS, MK) and interpretation of data (SvB, LAvdS, MK, FRR). All authors are involved in drafting the manuscript for intellectual content, and approved the final version. SvB and MK take responsibility for the integrity of the work as a whole.

## Declaration of competing interest

The Hostas study is financially supported by the Dutch 10.13039/501100000142Arthritis Society, who did not have influence on the design, performance and analysis of the study, nor on the decision to submit the manuscript for publication. SvB, LAvdS, FRR report no conflicts of interest. MK reports consultancy/lecture fees outside the submitted work from 10.13039/100004319Pfizer, 10.13039/100004336Novartis, 10.13039/100011110UCB, 10.13039/100012525Galapagos, 10.13039/100007091Flexion, 10.13039/100016492Kiniksa, 10.13039/100003898Janssen, 10.13039/100006483AbbVie, 10.13039/100018266CHDR, all paid to institution. Royalties from Wolters Kluwer and Springer Verlag, paid to institution.
