# Supplementary material for: Patients with clinically diagnosed hand OA not fulfilling the ACR classification criteria are in an earlier disease phase and more often have thumb base OA
Source: Osteoarthr Cartil Open. 2023 Feb 18;5(2):100347. doi: 10.1016/j.ocarto.2023.100347 (PMC10023912; doi:10.1016/j.ocarto.2023.100347)
Supplement: Multimedia component 1 [file mmc1.docx]

**Supplementary Figure 1** Cumulative probability plot showing the distribution of joints with hard tissue enlargement at baseline stratified for ACR^+^ (*blue diamond*) and ACR^−^ (*red dot*) patients.
