# Supplementary material for: Patients with clinically diagnosed hand OA not fulfilling the ACR classification criteria are in an earlier disease phase and more often have thumb base OA
Source: Osteoarthr Cartil Open. 2023 Feb 18;5(2):100347. doi: 10.1016/j.ocarto.2023.100347 (PMC10023912; doi:10.1016/j.ocarto.2023.100347)
Supplement: Multimedia component 2 [file mmc2.docx]

**Supplementary table 1** Baseline characteristics compared in the subgroup of baseline ACR^−^ patients (n=53) between patients newly fulfilling the criteria (ACR^+^) and patients still not fulfilling the criteria (ACR^−^) after 4 year follow-up

|  | **ACR^+^** | **(n=37)** | **ACR^−^** | **(n=16)** | ***p*=** |
| --- | --- | --- | --- | --- | --- |
| Age (years) | 56.6 | (7.2) | 59.4 | (10.1) | 0.264 |
| Women, n (%) | 28 | (75.7) | 15 | (93.8) | 0.250 |
| BMI (kg/m^2^) | 26.5 | (25.0–29.2) | 31.1 | (27.7–34.1) | 0.039 |
| Any comorbidity present, n (%) | 14 | (38.9) | 6 | (40.0) | 0.941 |
| Number of comorbidities | 0 | (0–1) | 0 | (0–1) | 0.704 |
| Generalized OA, n (%) |  |  |  |  |  |
| Knee and/or hip OA | 20 | (54.1) | 7 | (46.7) | 0.629 |
| Knee OA | 19 | (51.4) | 7 | (46.7) | 0.760 |
| Hip OA | 3 | (8.1) | 0 | (0.0) | 0.545 |
| **General and disease-specific burden** |  |  |  |  |  |
| Self-reported health-related quality of life |  |  |  |  |  |
| PCS | 41.8 | (36.8–48.8) | 42.3 | (35.5–48.9) | 0.982 |
| MCS | 54.7 | (48.8–57.0) | 54.7 | (49.3–60.8) | 0.580 |
| AUSCAN pain (0-20) | 10 | (6–13) | 9 | (6–13) | 0.857 |
| AUSCAN stiffness (0-4) | 2 | (1–2) | 2 | (1–3) | 0.667 |
| AUSCAN function (0-36) | 14 | (9–22) | 18 | (13–21) | 0.491 |
| AUSCAN total score (0-60) | 24 | (18–37) | 31 | (20–36) | 0.582 |
| **Fulfilment of each criterium from the ACR classification criteria at baseline** | | | | | |
| Major criterium (hand pain, aching or stiffness), n (%) | 31 | (83.8) | 15 | (93.8) | 0.661 |
| Minor criteria, n (%) |  |  |  |  |  |
| Hard tissue enlargement ≥ 2 of 10 selected joints* | 14 | (37.8) | 5 | (31.3) | 0.646 |
| Hard tissue enlargement ≥ 2 DIPJs | 13 | (35.1) | 4 | (25.0) | 0.468 |
| Swelling < 3 MCPJs | 36 | (97.3) | 16 | (100.0) | 0.999 |
| Deformity ≥ 1 of 10 selected joints* | 11 | (29.7) | 4 | (25.0) | 0.726 |
| **Hand-specific disease characteristics** |  |  |  |  |  |
| Radiography |  |  |  |  |  |
| Summated KL-scores (0-120) | 7 | (4–14.5) | 7 | (3–18) | 0.954 |
| KL-score ≥2 joint count (0-30) | 1 | (0–3) | 1 | (0.25–3.5) | 0.944 |
| HOA phenotype, n (%)†: |  |  |  |  | 0.500 |
| - No radiographic HOA | 19 | (51.4) | 7 | (43.8) | - |
| - Radiographic IPJOA, without TB involvement | 3 | (8.1) | 0 | (0.0) | - |
| - Radiographic TBOA, without IPJ involvement | 10 | (27.0) | 5 | (31.3) | - |
| - Radiographic HOA, affecting both TB and IPJ | 5 | (13.5) | 4 | (25.0) | - |

Mean (SD) or median (IQR) unless stated otherwise. *Ten selected joints are left and right DIP2, PIP2, DIP3, PIP3 and CMC1 joints. †Radiographic HOA was defined as a KL-score ≥2 of at least one CMC1 joint or at least two other hand joints. ACR, American College of Rheumatology; AUSCAN, Australian/Canadian osteoarthritis hand index; BMI, body mass index; CMC1, first carpal-metacarpal; DIP(J), distal interphalangeal (joint); HOA, hand osteoarthritis; KL-score, Kellgren-Lawrence osteoarthritis grading scale; MCPJ, metacarpal-phalangeal joint; MCS, mental component summary scale; PCS, physical component summary scale; PIP, proximal interphalangeal; TB, thumb base.
